# On Hepatic Congestion

**Published:** 1818-12

**Authors:** 


*For the London Medical and Physical Journal.*

### *On Hepatic Congestion.* By Dr. Kinglake.

THERE is, perhaps, no viscus in the human frame that is more importantly and critically connected with life and health than the liver. Its function, as a transmitting organ, is not less indispensable than its secreting office. The vast mass of venous blood that is incessantly passing through it, and the materials which it furnishes during its transit, for the formation of bile, render it of primary concern in estimating the diseased state of the chylopoïetic viscera. The large bulk of the liver, and its comparatively inirritable state, subject it to frequent incumbrances, and occasional obstructions, from the quantity of fluids destined to pass through it. The incipient stages of disease arising from undue vascular fullness of the liver, are not shown in symptoms that importunately crave either notice or assistance. The circumstance of the liver naturally possessing a low degree of excitability, permits real, and often serious, derangement of its healthy condition, without its becoming known by any corresponding sensation that could denote such commencement of mischief. A sense of weight and oppression about the hepatic region commonly announces the first departure from the healthy state; this feeling soon proceeds to a disordered condition of the digestive process, inducing nausea, loss of appetite, torpid bowels, either a deficient or excessive quantity of bile in the intestinal discharges, tumid abdomen, scanty and high-coloured urine, pallid and sometimes biliously-tinged countenance. These symptoms, either in part or collectively, evince a condition of disease that will most likely be progressive, without seasonable aid. It is not, indeed, in the nature of things that it should retrograde, and spontaneously attain a favourable termination. Diseased processes are either advanced or retarded by the greater or less excitability of the part in which they occur. In the heart, and arterial system generally, and in the brain, stomach, and intestines particularly, diseased action will assume an acute and rapid course; whilst, in the hepatic, the renal, and pulmonary structure, morbid occurrences will become less violent, and have a more tardy progress.

It is, in all cases of disease, most material to check their growth, and to limit within safe bounds their augmentation. Early and adequate means will be required for this purpose; and it is obvious that the most successful portion of the practice of medicine consists in repressing the originating stage of diseases, so that they may not acquire, by long continuance, either an habitual obstinacy or an insuperable establishment. Protracted disease, whatever may have been its original nature or situation, has a tendency to become irretrievably vitiated, and sooner or later irreparably to damage the structure in which it prevails. The liver, of all other viscera, is most prone to the insidious and irremediable evils of neglected or delayed disease; and, when the morbid injury to which it is liable has been fully induced, the prospect of benefit is as distant as the means for effecting it are visionary and insufficient.

The congestive fullness, which it is here more particularly the object to consider, is that in which the hepatic vessels, intended for the transmission of venous blood through the liver, are so surcharged as to impede the circulating current through them. The immediate effect of this obstruction is shown in the symptoms already recited; the ultimate result, in the detained blood gaining itself a passage through the biliary ducts into the duodenum, where it accumulates in large quantities, until discharged either by vomiting or purging. The experienced practitioner must have often seen the vast portions of sanguineous fluid that are occasionally evacuated in the way stated. From two to six quarts have been, in several instances, brought away, either by the action of vomiting or by intestinal evacuations, in the course of two or three days. The skin has been blanched, and arterial action has been diminished to a thread-like slenderness, by the continued and excessive drain through this unnatural route. But yet the patients have, in general, rallied, and have finally recovered, not only from the direct shock of losing so large a quantity of blood, but also from the lingering hepatic affection that had occasioned so inordinate a waste of the vital fluid. The discharged fluid appears, on examination, to be substantially, and for the most part, mere blood mixed with bilious and mucous substances. Its colour is dark, and its consistence grumous, with coagulated portions irregularly distributed through the mass. In pouring the fluid from one bason to another, bloody streaks, more or less fluid, are exhibited, especially at the bottom of the mass. This fluid, it may be presumed, is what the ancients denominated black-bile, and which is described as occurring at times in very large quantities. The venous plethora and vascular distension of liver, of which this fluid is the eventual product, must, in all periods, have been an occurring disease. The degrees of this affection must, of course, have been various, and its excessive instances only have attracted particular notice.

In most hepatic diseases, whether direct or indirect, there is a strong tendency to this unnatural escape of blood into the duodenum. In chronic hepatitis, in abdominal dropsy, and hæmorrhoidal obstructions, in dyspepsia, in low remittent fever, &c. any occurring surcharge of blood in the liver is apt to force itself a passage through the biliary ducts into the duodenum. Females are, agreeably to my own opportunities for observing, much more frequent subjects of this disease than males. It occurs, however, in both sexes, but it would appear much oftener in the former than in the latter. The complaint itself, abstractedly considered, is more alarming than really dangerous. As the effect of an existing cause, it commonly affords immediate and lasting relief. The deadly oppression that usually precedes its appearance is greatly diminished, if not wholly terminated, by its occurring; and the action of the heart and arteries acquire an improved freedom and power, by the removal of so unwieldy an obstacle to the circulating fluids. The blood that is lost on these occasions may be regarded, with reference to its late use in the circulating mass, as extravasated; at least, it is not directly abstracted from the sanguiferous vessels: it had been, for the most part, detained in an almost stagnant state in the vascular structure of the liver; its removal, therefore, was the cessation of an incumbrance that constituted a serious state of disease, and not a direct loss from the contents of the vessels that are carrying on the functions of life. Whenever this occurrence presents, it should be considered as a symptom indicating an advanced state of disease, that might have been restrained and prevented by a seasonable use of suitable remedies. But, viewing it as the result of accumulated obstruction, it may be justly regarded as more likely to benefit than to injure; and the discharges, frightful as they may appear, had better be promoted than checked. The exit by the bowels, after the deposition has taken place in the duodenum, is preferable to that by the gullet; it is less exhausting, and less likely to emulge and drain the liver to a hurtful extent, than the action of vomiting would be. The peristaltic power of the intestines should be excited by five grains of calomel, and ten of rhubarb, morning and evening, as long as the discharges may continue to be bloody; directing, in the interval, to dilute plentifully with thin oat-gruel.

When the stomach is capable of receiving, retaining, and digesting food, mild nutritive substances,—such as the yolk of egg, about a common size tea-cup full of warm milk at a time, light bread-pudding, mutton-broth, &c.—may be occasionally taken. Barley-water, slightly acidulated with muriatic acid, will prove a grateful and an efficacious tonic, and will in general be found preferable to a more varied and complex medicinal treatment.

It has not occurred to me to see a case of the kind in question in which bleeding has been required. No symptom of inflammatory excitement has attended; nor would the very enfeebled state of the vascular action admit of considering that mode of remedy at all applicable. But it is highly probable that the most essential preventive aid might be afforded by early general bleeding, and by sustaining such efficient action of the peristaltic power of the intestines as would sympathetically promote the biliary secretion, and thereby assist the liver in duly returning its venous blood into the general circulation. In addition to the evacuant influence of calomel on the intestines, its stimulant action on the general system, given in alterative doses night and morning, may salutarily excite the liver, and obviate the ultimate effects here stated, of morbid torpor, congestive fullness, and unyielding obstruction of that organ.

*Taunton; September 17th,* 1818.

